# Retinal Image Graph-Cut Segmentation Algorithm Using Multiscale Hessian-Enhancement-Based Nonlocal Mean Filter

**DOI:** 10.1155/2013/927285

**Published:** 2013-04-11

**Authors:** Jian Zheng, Pei-Rong Lu, Dehui Xiang, Ya-Kang Dai, Zhao-Bang Liu, Duo-Jie Kuai, Hui Xue, Yue-Tao Yang

**Affiliations:** ^1^Suzhou Institute of Biomedical Engineering and Technology, Chinese Academy of Sciences, Suzhou 215163, China; ^2^Department of Ophthalmology, The First Affiliated Hospital of Soochow University, Suzhou 215006, China; ^3^Institute of Automation, Chinese Academy of Sciences, Beijing 100190, China

## Abstract

We propose a new method to enhance and extract the retinal vessels. First, we employ a multiscale Hessian-based filter to compute the maximum response of vessel likeness function for each pixel. By this step, blood vessels of different widths are significantly enhanced. Then, we adopt a nonlocal mean filter to suppress the noise of enhanced image and maintain the vessel information at the same time. After that, a radial gradient symmetry transformation is adopted to suppress the nonvessel structures. Finally, an accurate graph-cut segmentation step is performed using the result of previous symmetry transformation as an initial. We test the proposed approach on the publicly available databases: DRIVE. The experimental results show that our method is quite effective.

## 1. Introduction

The retina is the only tissue in human body from which the information of blood vessel can be directly obtained in vivo. The information of retinal vessel plays an important role in the diagnosis and treatment of various diseases such as glaucoma [1], age-related macular degeneration [2], degenerative myopia, and diabetic retinopathy [3]. Recently, it is also found that the detection of vascular geometric change might be meaningful to judge whether people have high blood pressure or cardiovascular disease [4]. Retinal vessel extraction is quite essential for ophthalmologists to diagnose various eye diseases. Moreover, accurately segmented vessels could be very helpful for feature-based retinal image registration.

Generally, existing retinal vessel segmentation methods can be roughly divided into two categories. The first category is supervised learning-based method. Such methods require tremendous manual segmentations of vasculature to train the classifier. Staal et al. [5] first adopt a ridge-extraction method to separate the image into numerous patches. For each pixel in the patch, feature vector that contains profile information such as width, height, and edge strength is computed. Feature vectors are then classified using a *k* nearest neighbors classifier with sequential forward feature selection strategy. Soares et al. [6] selected pixel value and multiscale 2D Gabor wavelet coefficients to construct the feature vector. A Bayesian classifier based on class-conditional probability density functions was adopted to perform a fast classification and model complex decision surfaces. Marín et al. [7] proposed a new supervised method to segment retinal vessels. For each pixel, 5 gray-level descriptors and 2 moment invariants-based features of a squared window were computed as the feature vector. A multilayer neural network scheme was then adopted to classify each pixel. The performance of such method depends on the correlation between training data and test data. If these two datasets are quite different, the segmentation results may be less than ideal. Besides that, the manual segmentation step would bring additional burden to ophthalmologists. Therefore, such methods have not been widely used in clinic yet.

The second category is rule-based method. Such methods usually need to extract neighborhood information of each pixel. The information is then used to label the pixel according to some f preset rules. Matched filter [8], model-based method [9], and morphology-based method [10] all belong to this category. In the literature [8], Sofka and Stewart proposed a multiscale Gaussian and Gaussian-derivative profile kernel to detect vessels at a variety of widths. Lam et al. [9] proposed a multiconcavity modeling approach to handle unhealthy retinal images. In Lam's approach, a lineshape concavity measure was used to remove dark lesions and a locally normalized concavity measure is designed to remove spherical intensity variation. The two concavity measures are combined together according to their statistical distributions to detect vessels in general retinal images. Mendonça and Campilho [10] proposed a region growing algorithm in the morphological processed image to extract vascular centerline. First, four-direction-based differential operator was adopted to detect vascular centerlines. These centerlines were then merged to complete vascular centerlines and selected as seed points. Finally, region growing step was performed to reconstruct the vasculature. These methods do not require training steps and the interactions with doctors are also minimized; thus rule-based methods have been widely used in clinical application. However, ideal automatic retinal vessel segmentation is still not easy. This can mainly be ascribed to two reasons. One is that the contrast of some tiny vessels may be quite low, especially in some pathologies affected images. The other reason is that the image noise such as edge blurring may disturb the final segmentation results. 

Recently, graph-cut methods [11–15] have been very popular in image segmentation. This is because graph-cut methods could reach the global optimal value of the predefined energy function. Besides that, the user interactions of such methods are also very simple. Several graph-cut-based methods have been proposed to solve retina image segmentations. Chen et al. [16] proposed a 3D graph-search-graph-cut method to segment multilayers of 3D OCT retinal images. The multi-layers of retina and the symptomatic exudate-associated derangements (SEAD) are successfully segmented. Inspired by the superior performance of graph-cut method, we propose a new method to extract the blood vessels in retinal images following our previous work [17]. First, we perform a novel multiscale Hessian-based filter to compute the maximum response of vessel likeness function for each pixel, which is used to enhance the blood vessels of gray retinal images. Then, we adopt a nonlocal mean filter to suppress the noise of the enhanced image. After that, a radial gradient symmetry transformation is adopted to improve the detection of vessel structures and suppress the nonvessel structures. Finally, an accurate graph-cut segmentation is performed using previous symmetry transformation as an initial. 

## 2. Methods

### 2.1. Multiscale Hessian-Based Enhancement

In order to improve the contrast of retinal vasculature with different widths, we propose a multiscale Hessian-based enhancement. Frangi et al. [18] have proposed a method to detect the tubular structure based on the eigenvalues of Hessian matrix. We denote by *λ*_1,*s*_ and *λ*_2,*s*_ the eigenvalues of scale-related Hessian matrix, which is defined as follows:

(1) H(X,s)=[Ixx(X,s)Ixy(X,s)Iyx(X,s)Iyy(X,s)],

where *I*_*xx*_(*X*, *s*) is the second order differential of input image. For an ideal tubular structure, the eigenvalues generally will meet the following conditions:

(2) |λ1,s|≈0,|λ1,s|≪|λ2,s|.

We then define a new scale-related vessel likeness function:

(3) VL(X)=max⁡smin⁡<s<smax⁡V(s),

where *V*(*s*) is defined as follow:

(4) V(s)=|λ1,s|2·e|c−(|λ1,s|/λ1,s2+λ2,s2)|+|λ2,s|2·e|(|λ2,s|/λ1,s2+λ2,s2)−c|,

where *c* is an experiential parameter and we set it to $\sqrt{2}/2$.

**Figure 2:**
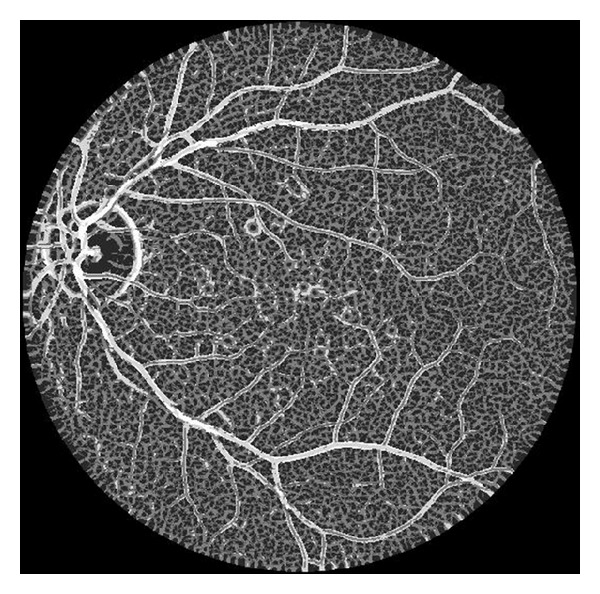
An example of the scale image of Figure 1; the pixel value stands for the scale that is corresponding to the maximal function value of *V*(*s*).

The function value of *V*(*s*) indicates the saliency of tubular structure for each pixel. We search in the scale range [*s*_min⁡_, *s*_max⁡_] to find the maximum response of vessel likeness function.  Figure 2 shows the optimal scale property of the input image. The pixel value stands for the scale that is corresponding to the maximal function value of *V*(*s*). As seen from Figure 2, the positive correlation between vessel width and optimal scale is obvious. The primary vessel owns a large scale property while the tiny vessel owns a small scale property.

**Figure 3:**
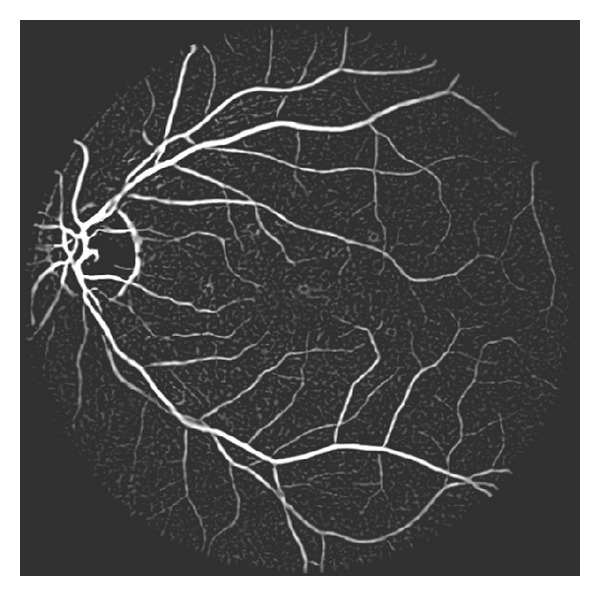
An example of the multiscale Hessian-based enhancement of Figure 1; the pixel value stands for the vessel likeness function value.

Figure 3 gives an example of multiscale Hessian-based enhancement, in which the pixel value stands for the vessel likeness function value. As shown in Figure 3, the whole retinal vasculature is prominently enhanced. However, some nonvessel structures are also enhanced including optic disk, yellow spots, and speckles. These nonvessel structures need to be removed in the following step.

### 2.2. Nonlocal Mean Filtering

**Figure 4:**
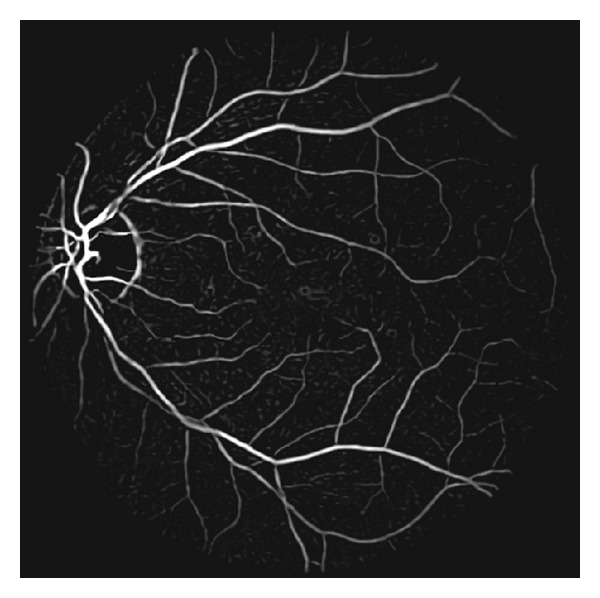
An output of the nonlocal mean filter of Figure 3, in which the image noise is effectively suppressed while the vasculature is maintained.

The effect of multiscale hessian based enhancement is obvious. As shown in Figure 3, the whole vasculature is significantly strengthened. However, some nonvessel structures are also enhanced and cause the enhanced image to be noisy. In order to suppress image noise and maintain the structural information, we employ a nonlocal mean filtering [19] step. We denote the enhanced image as VL(*X*) and the filtered image as NL(*X*). The detailed filtering step can be described as in the following equation:

(5) NL(i)=∑j∈Nw(i,j)·VL(j),

where *N* denotes the filtering neighborhood and *w*(*i*, *j*) is the weighting factor, which is defined as follow:

(6) w(i,j)=1Z(i)·exp⁡(−||v(Ni)−v(Nj)||2h2),Z(i)=∑jexp⁡(−||v(Ni)−v(Nj)||2h2),

where *v*(*N*_*i*_) denotes a feature vector which is composed of the pixel values of the nearby area around pixel *i* and *h* is a filtering parameter. The parameters of filtering neighborhood and nearby area need to be selected suitably so as to achieve a balance between filtering performance and computation cost.  Figure 4 shows a result of nonlocal mean filtering. We can find that the noise of enhanced image is suppressed effectively while the vasculature is maintained at the same time.

### 2.3. Radial Gradient Symmetry Transform

**Figure 5:**
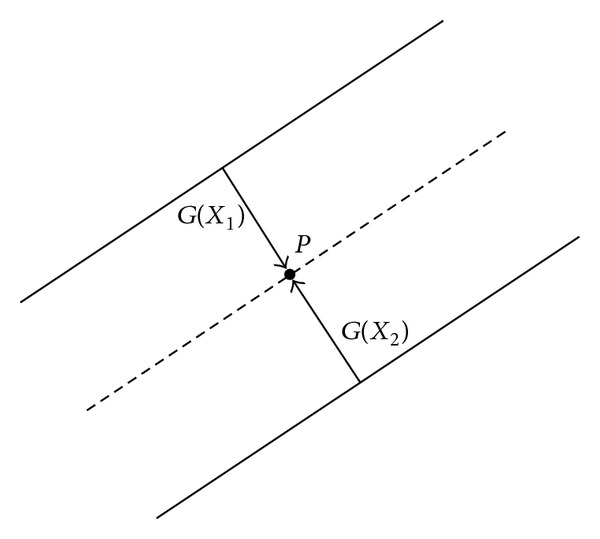
An example of radial gradient symmetry transform; we can find that the pixel in the vessel area is generally located between two symmetric gradient vectors.

In order to remove the nonvessel structures, we propose a radial gradient symmetry transform method based on Loy's work [20]. An ideal vessel structure is shown in Figure 5. As shown in Figure 5, we notice that the gradient vectors own a symmetric property in both the magnitude and direction. For those nonvessel structures, there is no such property. Therefore, we propose a symmetric vessel likeness function as follows:

(7) VLSym(X)=VLG(X)·FlagG(X),

where VL_*G*_(*X*) stands for a vessel likeness function of a given point *X* along the direction of gradient vector *G*(*X*) and Flag_*G*_(*X*) is an indicator function that indicates whether point *X* owns the gradient symmetric property. The computation of VL_*G*_(*X*) and Flag_*G*_(*X*) consists of the following steps.

(1) For each pixel in the filtered image as shown in Figure 4, we compute its vessel contribution along the gradient direction. The normalized gradient vector is denoted by *g*(*X*) = *G*(*X*)/||*G*(*X*)||. The coordinates of pixels that are affected by pixel *p* are computed as follows:
  (8) c(p)=p+round(r·g(p)),
 where *r* = 0, Δ*r*,…, 2 · *s*(*p*) and *s*(*p*) is the optimal scale parameter of pixel *p*, as shown in Figure 2.
(2) For these affected pixels *c*(*p*), we compute the vessel accumulation image *A* by the following equation:
  (9) A(c(p))=A(c(p))+2·s(p)−||c(p)−p||2·s(p).
(3) Then the VL_*G*_(*X*) is given by
  (10) VLG(X)=NL(X)·(AMn)q,
 where *M*_*n*_ is a normalization factor and *q* is a radial strictness parameter.
(4) For each pixel *p*, we search in its neighborhood [*p* − Δ*p*, *p* + Δ*p*] along the gradient direction with Δ*p* = 2 · *s*(*p*) · *g*(*p*). As shown in Figure 5, if there exist points *X*_1_ and *X*_2_ that meet the following condition, Flag_*G*_(*p*) = 1, otherwise; Flag_*G*_(*p*) = 0:
  (11) ||G(X1)||≥||G(p)||,  ||G(X2)||≥||G(p)||,g(X1)≈−g(X2).
(5) For symmetric vessel likeness function VL_Sym_(*X*), we set a vessel likeness threshold *T*_*h*_ to extract a coarse vasculature. After that, we take an erosion step to get a narrowed retinal vasculature. We then compute the pixel number of connected vessels. If the pixel number is smaller than the preset threshold *T*_num_, we consider it as the background noise and it should be removed.

### 2.4. Graph-Cut Step

Throughout the previous processing steps, we have got a coarsely extracted vasculature. The final step is to accurately segment vessels from previous results. This can be described as a pixel labeling problem which can be formulated using the energy function:

(12) E(L)=Ed(L)+kEs(L),

where *L* is a labeling set and *E*_*d*_(*L*) = ∑_*p*∈*P*_ *U*_*p*_(*l*_*p*_) is the data prior energy, which measures the cost of giving a label *l*_*p*_ ∈ *L* to a given pixel *p* according to prior information. *E*_*s*_(*L*) = ∑_*N*⊂*P*_∑_*q*∈*N*_ *V*_*pq*_(*l*_*p*_, *l*_*q*_) is the potential energy, which measures the smoothness of a neighboring pixel system *N* and *k* is a weighting parameter. 

We adopt the graph-cut algorithm to optimize the energy function (12). The centerline is used as shape prior to guide the extraction process. In our framework, a graph *G* = 〈*V*, *ε*〉 is created with nodes corresponding to pixels *p* ∈ *P* of a retinal image, where *V* is the set of all nodes and *ε* is the set of all links connecting neighboring nodes. The neighboring pixel system is constructed with eight neighboring pixels. The terminal nodes are defined as source *S* and sink *T*. As an initial, we extract the centerline of previously extracted vasculature. The pixels on the centerline are considered as definite foreground *F*_*d*_ and the pixels with VL_Sym_(*X*) > *T*_*h*_ are classified as candidate foreground *F*_*c*_. The pixels with VL_Sym_(*X*) < *T*_*l*_ are classified as background *B*_*d*_ and others are classified as the candidate background *B*_*c*_. That is, *L* = {*f*_*d*_, *f*_*c*_, *b*_*c*_, *b*_*d*_} and *V* = {*S*, *T*}∪{*F*_*d*_, *B*_*d*_}∪{*F*_*c*_, *B*_*c*_}.

For each pixel *p* ∈ {*F*_*c*_, *B*_*c*_}, we compute its minimum distances to *S* and *T* according to the literature [22], which are denoted as *d*_*f*_(*p*) and *d*_*b*_(*p*), respectively. The cost of *t*-links can be computed as follows:

(13) US(p)=∞, UT(p)=0, p∈Fd,US(p)=0, UT(p)=∞, p∈Bd,US(p)=w1·VLSym(p)DF(p), UT(p)=w2·VLSym(p)DF(p),                     p∈Fc,US(p)=w2·VLSym(p)DF(p), UT(p)=w1·VLSym(p)DF(p),                     p∈Bc,

where *D*_*F*_(*p*) = *d*_*f*_(*p*)/(*d*_*f*_(*p*) + *d*_*b*_(*p*)), *D*_*B*_(*p*) = 1 − *D*_*F*_(*p*), and *w*_1_ > 1 > *w*_2_ > 0. The nodes in *F*_*d*_ and *B*_*d*_ are definitely labeled as *f*_*d*_ and *b*_*d*_, respectively. The weight of *n*-link describes the labeling coherence of a pixel with its neighbors. We utilize the pixel value information as the neighborhood penalty item. The cost of *n*-links can be defined as follows:

(14) Vpq(lp,lq)=1|I(p)−I(q)|+η,

where *η* is a tiny number to avoid division by 0. When the graph-cut algorithm terminates, we encourage candidate foreground pixels to be labeled as foreground and discourage candidate background to be classified as background pixels.

## 3. Experiments and Conclusion

We test our method on the publicly available databases: DRIVE [5]. Three measures sensitivity (SE), specificity (SP), and accuracy (AC) are used to evaluate the performance of our method in the image field of view. They are defined as follows:

(15) SE=N_correct_vesselN_vessel,SP=N_correct_nonvesselN_nonvessel,AC⁡=N_correct_totalN_total,

where *N*_correct_vessel is the number of correctly classified vessel pixels and *N*_vessel is the number of the vessel pixels in ground truth. *N*_correct_nonvessel is the number of correctly classified nonvessel pixels and *N*_nonvessel is the number of nonvessel pixels. *N*_correct_total is the total number of correctly classified pixels and *N*_total is total number of pixels.

We test the proposed method on 40 images and compare it with the methods developed by Staal et al. [5], Mendonça and Campilho [10], Wang et al. [21], and Marín et al. [7]. The mean values and standard deviations (sd.) of these methods are shown in Table 1. Some values that cannot be obtained from the literature are denoted by NA.

From Table 1 we can see that there is a prominent improvement in sensitivity. This means that our method is quite effective in extracting some tiny vessels. On the other hand, our method is not as well as others' in both specificity and accuracy. For clinical application, the accuracy should be better than 90%. Our method is over the qualified standard, but there are still large improvements need to be done. 

**Figure 6:**
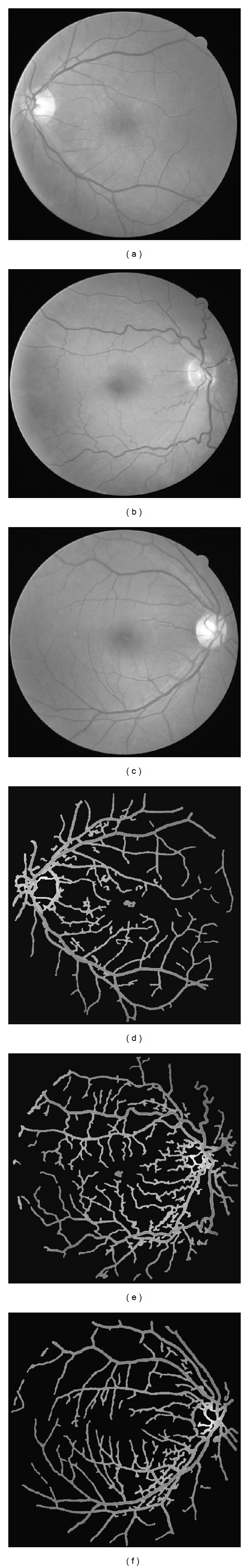
An example of 3 extraction results: the first row shows 3 input retinal images and the second row shows the segment results of the retinal vessels.

**Figure 7:**
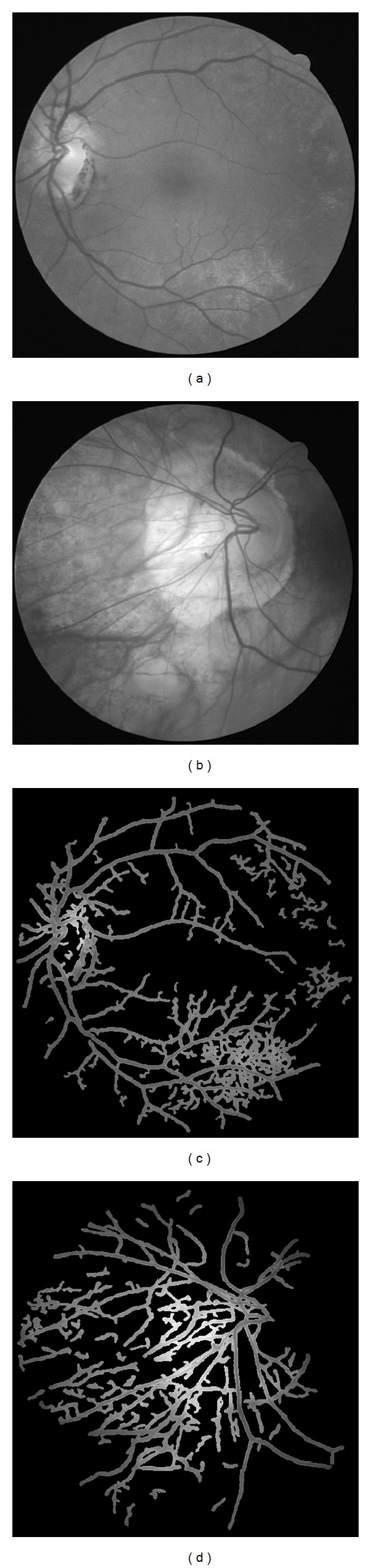
Two segmentation results of ill-conditioned retinal images; the speckles greatly reduce the performance of our algorithm.

Some of the extraction results are shown in Figures 6 and 7. As it can be seen from Figure 6, the majority of the vessels in the retina can be finely extracted; however, some nonvessel structures are also extracted and some tiny vessels are not correctly segmented. This is mainly because our method focused more on tiny vessel extraction, which is not easy for ophthalmologists to extract manually. Therefore some boundaries between retinal region and the background are mistakenly recognized as vessels. Besides that, the optic disk also cannot be fully excluded from the final result.  Figure 7 shows two segmentation results of ill-conditioned retinal images, where some speckles are hard to be differentiated from tiny vessels. Our method fails to extract an accurate vasculature, where the AC values are 0.8720 and 0.8814, respectively. Furthermore, we exchange the order of nonlocal mean filtering and multiscale hessian-based enhancement. Experimental results show that there is a little difference between the performances. This also indicates that nonlocal mean filter is very robust and could be widely used in image processing area. 

In summary, this paper first presents a multiscale hessian-based enhancement for retinal images. Next, we adopt an effective nonlocal mean filtering step to suppress noise of the enhanced image. Then, we propose a radial gradient symmetry transform method to suppress the nonvessel artifacts. Finally, a graph-cut step is taken to accurately segment the retinal vessels. Experiments show that our method is very sensitive for the vessels segmentation, but the performance for some tiny vessel extraction and speckles exclusion is still needed to be improved. This will be our further work. We will make further studies to improve the performance.

## Figures and Tables

**Figure 1:**
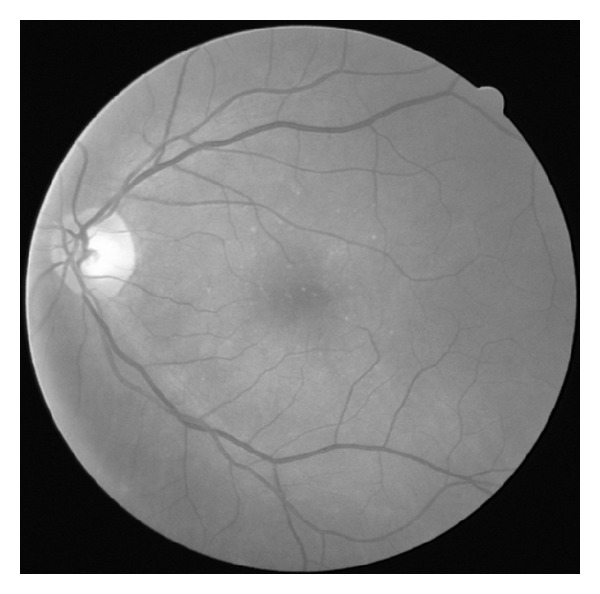
An example of an input retinal image, downloaded from publicly available databases: DRIVE (http://www.isi.uu.nl/Research/Databases/DRIVE/).

**Table 1:** Comparison of different segmentation methods.

STARE	SE (mean/sd.)	SP (mean/sd.)	AC (mean/sd.)
Staal et al. [5]	0.7194/0.0694	0.9773/0.0087	0.9441/0.0065
Mendon*ç*a and Campilho [10]	0.7315/NA	0.9781/NA	0.9463/NA
Wang et al. [21]	0.7810/0.0340	0.9770/0.0071	NA
Marín et al. [7]	0.7067/0.0628	0.9801/0.0104	0.9452/0.0064
Ours	0.9074/0.0332	0.9119/0.0320	0.9113/0.0280
